# Prognostic Value of C-Reactive Protein, Leukocytes, and Vitamin D in Severe Chronic Obstructive Pulmonary Disease

**DOI:** 10.1155/2014/140736

**Published:** 2014-01-21

**Authors:** Mia Moberg, Jørgen Vestbo, Gerd Martinez, Peter Lange, Thomas Ringbaek

**Affiliations:** ^1^Section of Respiratory Medicine, Hvidovre University Hospital, Kettegaard Alle 30, 2650 Hvidovre, Denmark; ^2^Department of Respiratory Medicine, Odense University Hospital, University of Southern Denmark, Sdr. Boulevard 29, 5000 Odense C, Denmark; ^3^Respiratory and Allergy Research Group, Manchester Academic Health Science Centre, University Hospital of South Manchester NHS Foundation Trust, Southmoor Road, Manchester M23 9LT, UK; ^4^Section of Social Medicine, Department of Public Health, University of Copenhagen, Øster Farimagsgade 5, 1014 Copenhagen K, Denmark

## Abstract

Inflammatory biomarkers predict mortality and hospitalisation in chronic obstructive pulmonary disease (COPD). Yet, it remains uncertain if biomarkers in addition to reflecting disease severity add new prognostic information on severe COPD. We investigated if leukocytes, C-reactive protein (CRP), and vitamin D were independent predictors of mortality and hospitalisation after adjusting for disease severity with an integrative index, the i-BODE index. In total, 423 patients participating in a pulmonary rehabilitation programme, with a mean value of FEV_1_ of 38% of predicted, were included. Mean followup was 45 months. During the follow-up period, 149 deaths (35%) were observed and 330 patients (78.0%) had at least one acute hospitalisation; 244 patients (57.7%) had at least one hospitalisation due to an exacerbation of COPD. In the analysis (Cox proportional hazards model) fully adjusted for age, sex, and i-BODE index, the hazard ratio for 1 mg/L increase in CRP was 1.02 (*P* = 0.003) and for 1 × 10^9^/L increase in leukocytes was 1.43 (*P* = 0.03). Only leukocyte count was significantly associated with hospitalisation. Vitamin D was neither associated with mortality nor hospitalisation. Leukocytes and CRP add little information on prognosis and vitamin D does not seem to be a useful biomarker in severe COPD in a clinical setting.

## 1. Introduction

Chronic obstructive pulmonary disease (COPD) is an inflammatory lung disease associated with significant systemic consequences [1]. Several biomarkers have been investigated in search of a better tool to predict clinically relevant outcomes such as mortality, hospitalisation, and exacerbations [2]. Biomarkers such as C-reactive protein (CRP) and leukocytes represent low-grade systemic inflammation and increased levels have been found in patients with COPD [3] and have been associated with a poor prognosis [4] and comorbidity [5]. Vitamin D on the other hand has anti-inflammatory properties and vitamin D deficiency has been associated with all-cause mortality in the general population [6] and mortality specifically linked to diseases of the respiratory system [7]. Vitamin D deficiency is common in patients with COPD [8], but the prognostic value of vitamin D has only to a lesser extent been investigated in this particular group of patients.

FEV_1_ (forced expiratory volume in 1 second) is considered an important and valuable marker of disease severity but is less appropriate to characterise disease activity and predict progression [9]. Despite the clear association between inflammatory biomarkers and COPD, it still remains uncertain whether these biomarkers, like FEV_1_, only reflect disease severity or whether they add new information in addition to clinical measures of the disease. In order to further investigate this matter, we hypothesised that CRP, leukocytes, and vitamin D were independent predictors of mortality and hospitalisation for COPD exacerbation and that their predictive value would persist after adjusting for disease severity with an integrative index, the i-BODE index. The i-BODE is a modified version of the BODE-index [10]. The original BODE-index has been found to predict risk of mortality [11], hospitalisation [12], and COPD exacerbations [13].

The purpose of the present study was to assess the prognostic value of CRP, leukocytes, and vitamin D level in a cohort of patients with severe COPD referred for outpatient rehabilitation.

## 2. Methods

### 2.1. Selection of Patients

The patients included in the present study participated in a seven-week pulmonary rehabilitation programme at Hvidovre Hospital, Copenhagen, during the period of May 2005 to March 2011. The main criteria for referral to the rehabilitation programme were stable COPD, FEV_1_ < 50% of predicted, and MRC (Medical Research Council) dyspnoea scale grade ≥3; nevertheless, all patients were assessed individually in terms of suitability for participation.

### 2.2. Incremental Shuttle Walking Test (ISWT) and i-BODE Index

ISWT was measured at baseline using the protocol described by Singh et al. [14]. Variables and point allocation used for the computation of the i-BODE index are shown in Table 1 and were validated in a study by Williams et al. [10]. The original BODE index consists of body mass index (BMI), airflow obstruction as measured by FEV_1_% predicted, functional dyspnoea as measured by the mMRC (modified Medical Research Council) dyspnoea scale, and exercise capacity as measured by the 6-minute walking test [11]. In the i-BODE index, the 6-minute walking test is substituted with the ISWT as a measurement of exercise capacity and the mMRC dyspnoea scale has been replaced with the MRC dyspnoea scale (grades 1–5) [10].

### 2.3. Laboratory Measurements

The patients had CRP, leukocytes, and vitamin D measured at the time they entered the rehabilitation programme. All blood samples were analysed at Hvidovre Hospital shortly after they were drawn. The department of Clinical Biochemistry, Hvidovre Hospital, used two different methods for CRP and leukocytes during the study period (2005–2011). Plasma CRP was measured on a Vitros FS 5.1 (Ortho Clinical Diagnostics, Rochester, NY, USA) during the first half of the study period and on a Cobas 6000 (c501) module (Roche Diagnostics GmbH, Penzberg, Germany), in the second half, both according to the manufacturers' specifications and using proprietary reagents. Leukocytes were counted by using the ADVIA 120 Hematology Analyzer System (Bayer, Holliston, MA, USA) in the first half of the study period and Sysmex XE-5000 (Sysmex Corporation, Japan) in the second half. Measurement of 25(OH)D was conducted by means of a direct, competitive chemiluminescence immunoassay using the DiaSorin LIAISON 25(OH)D Total assay (DiaSorin, Inc., Stillwater, MN, USA). The assay measured both 25-hydroxyvitamin D_3_ and 25-hydroxyvitamin D_2_.

### 2.4. Mortality and Hospitalisation

The National Health Services Central Register ascertained vital status and provided information on all hospital admissions in the follow-up period up to 24 January, 2012.

A primary diagnosis of COPD (J44.x), or a primary diagnosis of respiratory failure (J96.x) with a secondary diagnosis of COPD (J44.x) at discharge, was recorded as an admission due to exacerbation of COPD. The diagnoses were made according to the 10th edition of the International Classification of Diseases (ICD). The Danish Data Inspection Board has approved the present study.

### 2.5. Statistics

Cox proportional hazards model was used to identify factors that significantly predicted mortality and time to first hospitalisation. Variables included in the model were age, sex, CRP, leukocyte count, vitamin D, FEV_1_, and i-BODE index score. Assumption of linearity was assessed by categorising the variable into multiple dichotomous variables of equal units (quartiles) on the variable's scale. The results of the regression analyses are given in terms of estimated hazard ratios with corresponding 95% confidence intervals (CI).

CRP, leukocyte count, and vitamin D were the variables of interest. They were entered into the model as dichotomous as well as continuous variables, first in a univariate analysis and then in a multivariate analysis adjusting first for age and sex, then age, sex, and FEV_1_% predicted, and lastly age, sex, and i-BODE index. When smoking status was added to the model, the *P*  values did not change significantly. The cut-off values for the dichotomous values were as follows: CRP < 10 mg/L, leukocytes ≤ 8.8 × 10^9^/L, and vitamin D ≤ 50 nmol/L.

We also combined the dichotomous values into two new variables in which all patients with either two or three biomarkers outside the normal range (the cut-off values are seen above) were grouped. This was similar to the method used in previous studies [15, 16] and was done in order to see if it was possible to identify patients with increased risk of all-cause mortality and hospitalisation.

Kaplan-Meier survival curves were created for descriptive purposes using the statistical package for the social sciences (SPSS) version 20.0 (SPSS Inc., Chicago, IL, USA). A *P*  value <0.05 was considered statistically significant.

We used chi-squared test for binary variables and an independent *t*-test for continuous variables when comparing baseline characteristics of the study population and the excluded population, that is, patients with missing data. The characteristics have been summarised as mean and standard deviation (SD) or percentage. CRP, leukocyte count, and vitamin D have been summarised as median and 25 and 75 percentiles.

## 3. Results

**Figure 1:**
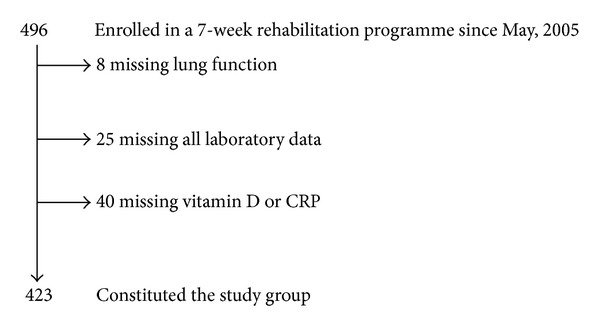
Flow chart of patients included in the study.

Our study group consisted of 423 consecutive patients as shown in Figure 1.

In general, most of the patients had severe airflow obstruction; 83.9% of the patients had FEV_1_ ≤ 50% predicted.  Table 2 compares baseline demographics of the study group and patients with missing laboratory tests.

In the study group, 208 patients (49.2%) were vitamin D deficient (25(OH)D less than 50 nmol/L), 197 (46.6%) had elevated leukocyte count (above 8.8 × 10^9^/L), and 102 (24.1%) had elevated CRP (above 10 mg/L). In total, 117 (27.6%) had two out of these three biomarkers outside the normal range. Only 38 (9%) had three out of three biomarkers outside the normal range.

Biomarkers were only weakly associated with COPD characteristics such as FEV_1_. Conversely, CRP and leukocyte count were positively correlated, and CRP was also positively correlated with BMI and negatively correlated with walking distance and desaturation during exercise. CRP was, however, not correlated with the i-BODE index. Leukocyte count was positively correlated with SGRQ (St. George's Respiratory Questionnaire) scores (higher scores indicate more limitations) and prevalence of heart disease.

During the follow-up period, 149 deaths (35.2%) were observed and a total of 330 patients (78.0%) had at least one acute hospital admission for any cause; 244 patients (57.7%) had at least one hospital admission due to an exacerbation of COPD. The hospital admissions for any cause were not dominated by any particular diagnoses.

### 3.1. Biomarkers and Mortality

**Figure 2:**
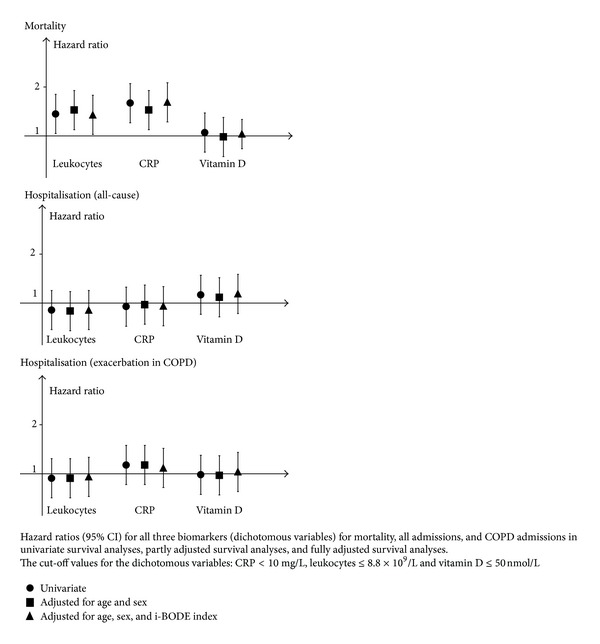
Hazard ratios for leukocytes, CRP, and vitamin D.

In the univariate analyses of the biomarkers as continuous variables, only CRP was significantly associated with mortality. In the analysis fully adjusted for age, sex, and i-BODE index, the hazard ratio for 1 mg/L increase in CRP was 1.02 (95% CI: 1.01–1.04, *P* = 0.003). Leukocyte count and vitamin D as continuous variables were not significantly associated with mortality. When entering biomarkers as dichotomous variables in the models, both leukocyte count and CRP were significant predictors of mortality both in the univariate and the adjusted analyses (Figure 2). Two out of three biomarkers outside the normal range predicted mortality both in the univariate and the multivariate analyses with a hazard ratio of 1.48 (95% CI: 1.06–2.08, *P* = 0.02) and 1.50 (95% CI: 1.07–2.10, *P* = 0.02), respectively. Three out of three biomarkers outside the normal range did not predict mortality.

### 3.2. Biomarkers and Hospitalisation

In the univariate analysis, leukocyte count (continuous variable) was significantly associated with hospitalisation. In the analysis fully adjusted for age, sex, and i-BODE index, the hazard ratio for leukocytes was 1.06 (95% CI: 1.02–1.10, *P* = 0.007) and 1.06 (95% CI: 1.01–1.11, *P* = 0.018) for hospitalisation due to any cause and hospitalisation due to exacerbation of COPD, respectively. CRP and vitamin D were not significantly associated with hospital admission and neither was the variable of two of three or three out of three biomarkers outside the normal range.

### 3.3. The i-BODE Index

The i-BODE index was a significant predictor of death and hospitalisation. The hazard ratio for death per one point increase in i-BODE score was 1.35 (95% CI: 1.23–1.49, *P* < 0.001). The hazard ratio for any acute hospitalisation per one point increase in i-BODE score was 1.16 (95% CI: 1.09–1.23, *P* < 0.001) and for hospitalisation with an exacerbation of COPD 1.24 (95% CI: 1.15–1.33, *P* < 0.001). We did not find interaction between variables in the adjusted analysis (age, sex, FEV_1_, and i-BODE index) and cardiac comorbidity.

## 4. Discussion 

In this study of patients with severe COPD, the prognostic value of three biomarkers was examined. After adjusting for the i-BODE index, we found that elevated leukocyte count was a significant predictor of both mortality and hospital admission, whereas elevated CRP levels only predicted mortality. Vitamin D status and three out of three biomarkers outside the normal range were associated neither with mortality nor hospitalisation. Two out of three biomarkers outside the normal range predicted mortality. As expected, the i-BODE index was a strong predictor of mortality and hospital admission, but none of the investigated biomarkers were associated with the i-BODE score. The results suggest that these biomarkers mostly express disease severity and did not add further information about prognosis in this sample of patients with severe COPD.

Our results regarding leukocytes and CRP are similar to those of previous studies. In the ECLIPSE (Evaluation of COPD Longitudinally to Identify Predictive Surrogate Endpoints) study, the association between leukocytes and mortality in stable COPD was investigated; adjusting for age, previous hospitalisations, and BODE index, leukocyte count was independently associated with mortality [17]. A cross-sectional study of patients with moderate to severe COPD also showed that elevated levels of leukocytes were associated with high resource utilisation in a multivariate analysis [18]. We found that levels of CRP were not associated with the i-BODE index. This is in line with two studies of patients with stable COPD in which the original BODE index was used [19, 20]. In the latter study by Liu et al., CRP and the BODE index were both independent prognostic variables for mortality and it seemed that the combination of the two variables predicted mortality better than either variable alone [20]. In a study using data from the Copenhagen City Heart Study and the Copenhagen General Population Study, the authors concluded that simultaneously elevated levels of high-sensitivity CRP, fibrinogen and leukocytes count in individuals with COPD were associated with increased risk of exacerbation [15]. This was also the case in milder disease and in those without a history of frequent exacerbations implying that the biomarkers added information beyond known clinical parameters. In some subgroups, the risk of exacerbation increased for every additional elevated biomarker. We could not show this additive effect. Most of the individuals in this prospective cohort were GOLD grade I-II and A-B; in contrast, almost all patients in the present study had more severe COPD being GOLD grade III–VI and D–B, as they had been selected for pulmonary rehabilitation. The fact that one of the biomarkers, vitamin D, did not have any predictive value also has to be taken into consideration.

The association between CRP and mortality was not very strong in our material, but in other studies CRP has been associated with mortality in patients with mild to moderate COPD [4, 21, 22]. This association could not be reproduced in patients with moderate to severe COPD [23], and even though the level of CRP is increased in patients with COPD compared with controls [24], it is an unspecific acute phase reactant and displays a wide variability in stable subjects with COPD over three months [25], which makes it less appropriate as a prognostic marker in this stratum of COPD.

We did not find that vitamin D predicted mortality in patients with COPD. This is in concordance with a recent prospective study in which 462 patients with moderate to very severe COPD were followed for 10 years [26]. However, several observational studies have shown that low vitamin D status predicts poor survival in the general population and non-COPD patients [6, 27, 28]. Intervention studies are called for as vitamin D deficiency is frequent in patients with severe COPD [8, 29] and has also been associated with low FEV_1_ [30] and respiratory tract infections [31]. Nevertheless, no effect of vitamin D supplementation was seen in COPD exacerbations in a randomised controlled study by Lehouck et al. except in a subgroup of patients with vitamin D status <10 ng/mL (25 nmol/L) [32]. Vitamin D levels may be low in patients with COPD and in general in patients with noncommunicable diseases [33], but it seems that vitamin D deficiency is not a major contributor to disease mechanisms or prognosis. Vitamin D deficiency may only be a marker of poor health in general but not specifically in COPD.

The patients in our study were well characterised and no patients were lost during followup; the patient cohort was very homogeneous because of the inclusion criteria for the rehabilitation programme. A weakness of the study was that we did not know whether the patients had been supplemented with vitamin D or not. We did not measure high-sensitivity CRP; however, regular CRP is readily available for everyone in daily clinical practice.

The main clinical implication of our finding is that even though epidemiological studies show that CRP and leukocytes may have a prognostic value in mild to moderate COPD, clinical characterisation of patients with severe COPD is often sufficient for prediction of clinical prognosis. None of the biomarkers seem to reflect disease activity. Leukocyte count and CRP add only little information on prognosis and vitamin D does not appear to be a useful biomarker in severe COPD.

## Figures and Tables

**Table 1:** Variables and point allocation used for the computation of the i-BODE index.

Variable	0 points	1 point	2 points	3 points
FEV_1_ predicted	≥65	50–64	36–49	≤35
Distance walked on ISWT (m)	≥250	150–249	80–149	<80
MRC dyspnoea grade	1-2	3	4	5
BMI (kg/m^2^)	>21	≤21		

**Table 2:** Characteristics of patients in the study group compared with excluded patients.

Group of patients	Study group	Excluded	*P* level
*N*	423	73	—
Females, %	61.0	70.0	0.15
Age, years	68.9 (9.3)	70.5 (10.2)	0.18
Pack years	40.6 (20.8)	44.7 (23.3)	0.13
Current smoking, %	26.8	20.5	0.26
Heart disease, %	46.8	45.2	0.80
Education, years	8.8 (2.5)	9.2 (2.7)	0.25
Lived alone, %	54.3	60.3	0.34
Examined in the winter period, %	75.1	77.0	0.76
FEV_1_, % predicted	37.7 (13.8)	38.9 (14.9)	0.55
Prednisolone treatment, %	3.1	6.8	0.11
Long-term oxygen therapy, %	5.0	6.8	0.51
Oxygen saturation at rest, %	94.5 (2.1)	94.4 (2.0)	0.64
Oxygen desaturation during SWT > 4%, %	218 (51.5)	32 (43.8)	0.22
SWT, metre	184.5 (95.7)	180.5 (92.9)	0.74
MRC dyspnoea score	4.0 (0.9)	3.8 (1.0)	0.05
BMI, kg/m^2^	26.0 (6.0)	26.0 (6.5)	0.99
Admissions to hospital the previous year^1,2,3^	0 (0, 1)	0 (0, 2.25)	0.79
Bed days the previous days^1,2,3^	0 (0, 5.25)	0 (0, 0)	0.23
i-BODE index, points	6 (3)	5.5 (2.2)	0.26
Vitamin D, nmol/L^2^	51 (29, 70)	—	—
CRP, mg/L^2^	6 (4, 10)	—	—
Leukocytes, 10^9^/L^2^	8.7 (7.3, 10.5)	—	—

^1^Due to exacerbation of COPD.

^2^Mean (25, 75) percentiles.

^3^Mann-Whitney *U* test.

SWT: Shuttle walking test.

MRC: Medical Research Council.

BMI: body mass index.
